# Applying Serum Proteins and MicroRNA as Novel Biomarkers for Early-Stage Cervical Cancer Detection

**DOI:** 10.1038/s41598-020-65850-z

**Published:** 2020-06-03

**Authors:** Shengye Du, Yinghui Zhao, Changyu Lv, Meiling Wei, Zheng Gao, Xianhua Meng

**Affiliations:** Department of Obstetrics and Gynecology, Jinan People’s Hospital Affiliated to Shandong First Medical University, No. 001 Xuehu Street at Northern Changshao Road, Laiwu District, Jinan, Shandong 271199 China

**Keywords:** Predictive markers, Cancer screening

## Abstract

Recently, we have been seeing emerging applications of non-invasive approaches using serum biomarkers including miRNA and proteins in detection of multiple cancers. Currently, majority of these methods only use solitary type of biomarkers, which often lead to non-satisfactory sensitivity and specificity in clinical applications. To this end, we established a unique biomarker panel in this study, which determined both squamous cell carcinoma antigen (SCC Ag) degree and miRNA-29a, miRNA-25, miRNA-486-5p levels in blood for detection of early-stage cervical cancer. We designed our study with two phases: a biomarker discovery phase, followed by an independent validation phase. In total of 140 early-stage cervical cancer patients (i.e., AJCC stage I and II) and 140 healthy controls recruited in the biomarker discovery phase, we achieved sensitivity of 88.6% and specificity of 92.9%. To further assess the predictive power of our panel, we used it to an independent patient cohort that consisted of 60 early-stage cervical cancer individuals as well as 60 healthy controls, and successfully achieved both high sensitivity (80.0%) and high specificity (96.7%). Our study indicated combining analyses of multiple serum biomarkers could improve the accuracy of non-invasive detection of early-stage cervical cancer, and potentially serve as a new liquid biopsy approach for detecting early-stage cervical cancer.

## Introduction

For women worldwide in the world, cervical cancer, which is ranked as the fourth most frequently occurred cancer^1^, contributed for 6.6% of the total cases of cancer and 7.5% of the total cancer fatalities of women in 2018^1^. Additionally, in women of reproductive age, cervical cancer is the major cause^2–4^. The transition to invasive cervical cancer from normal epithelium can take more than a decade^5^. In the early stage of Ib and II, the overall survival rate of cervical cancer is 70–90%, but this rate significantly goes down to 15% in the late stage of IVa^6^. The main reason of the high death rate of cervical cancer is that its asymptomatic and non-specific nature in the early stages makes early detection extremely difficult^7^. If early detection of cervical cancer is achieved, there are various treatment options readily available, which make cervical cancer curable.

Currently, Papanicolaou test (Pap smear) and colposcopy are the most common methods for cervical cancer detection. For pap smear screening, it had specificity of 98%, but a sensitivity of 51%^8^. Also, Pap smear is not very effective at identifying adenocarcinoma, or cervical carcinoma *in situ*. For certain early-stage cervical cancer, colposcopy and cervical biopsy may be able to recognize it, but these procedures are invasive for patients, and could delay treatment and generate extra costs and risks^9^.

Serum tumor biomarkers such as Carcinoembryonic antigen (CEA), squamous cell carcinoma antigen (SCC Ag) as well as CA19-9 have been frequently used for detecting and monitoring cervical cancer, because they can be measured non-invasively in blood samples^10–14^. However, none of them are specific enough to detect cervical cancer, or sensitive enough for early-stage cervical cancer detection^10,12–14^. Recently, microRNAs (miRNAs) were also suggested as encouraging biomarkers for non-invasive detection of cervical cancer with high sensitivity and specificity^15,16^. miRNAs are non-coding RNAs with 19–24 nucleotides. miRNAs were found to be frequently deregulated in cancer^17–19^. In recent studies, eight miRNA biomarkers, namely miRNA-20a, miRNA-205, miRNA-218, miRNA-21, miRNA-29a, miRNA-200a, miRNA-25, miRNA-486-5p, were discovered to be capable of discriminating cervical cancer patients with healthy controls^9,20–22^. Zhao *et al*. suggested that miRNA-20a level was elevated in the serum of cervical cancer patients, which was useful for detecting lymph-node metastasis^20^. Ma *et al*. observed that increase of miRNA-205 level in serum were related to cervical cancer tumor stage^21^. Tang *et al*. reported the expression levels of miRNA-218 decreased in serum as well as in cervical cancer tissue, which were associated with tumor stage in patients^22^. Jia *et al*. discovered that five miRNAs, namely miRNA-21, miRNA-29a, miRNA-200a, miRNA-25, and miRNA-486-5p, were up-regulated in patients with cervical cancer^9^.

While these pioneering studies successfully identified potential biomarkers to differentiate healthy controls from cervical cancer patients at early stages, these biomarkers were often evaluated individually and thus leading to a non-satisfactory diagnosis performance, i.e., low specificity and low sensitivity. Recently, several studies^23,24^ suggested that the combination of various analytes in blood dramatically improved the accuracy. For example, by analyzing multiple types of biomarkers including ctDNA mutations and serum proteins, CancerSEEK was able to detect multiple cancers and achieved a diverse range of sensitivity, i.e., from 98% in ovarian cancers to 33% in breast cancers^24^. In this study, we were encouraged by the previous successes on using combined analysis of multiple analytes for cancer detection, and aimed to develop a panel of biomarkers that could combine the usage of both miRNA and proteins for detecting cervical cancer at early stages. To this end, we first recruited a total of 140 early-stage cervical cancer patients as well as 140 healthy controls to discover the panel of biomarkers (i.e., the initial biomarker discovery phase). We evaluated the expression levels of miRNA-20a, miRNA-205, miRNA-218, miRNA-21, miRNA-29a, miRNA-200a, miRNA-25, miRNA-486-5p and protein levels of SCC Ag, CA19-9 and CEA in serum samples. Among these biomarkers, we chose three miRNAs, namely miRNA-29a, miRNA-25, miRNA-486-5p, and one protein biomarker, namely SCC Ag, as our panel of biomarkers for further investigation, due to their high value of area under curve (AUC) of the receiver-operating characteristic (ROC) curve. We found that using this panel of biomarkers, we could discriminate early-stage cervical cancer patients from healthy controls, achieving high sensitivity of 88.6% and high specificity of 92.9%. Next, we further evaluated the clinical application of our panel by applying it to an independent clinic study (i.e., validation phase). The validation phase of study included 60 early-stage cervical cancer patients and 60 healthy controls. We found that our panel of biomarkers could successfully separate early-stage cervical cancer patients from healthy controls at sensitivity of 80.0% and specificity of 96.7%. These studies confirmed that the panel of miRNA and protein could serve as an accurate, non-invasive approach for detection of early-stage cervical cancer. To our best knowledge, our study represents the first attempt of using both miRNA and protein biomarkers in non-invasive detection of cervical cancer at early stages.

## Results

### Overview of the design of this study

In brief, we designed this study with two phases, i.e., a biomarker discovery phase and a following independent validation phase. We aimed to discover a panel of serum biomarkers in the phase of the discovery study, followed by establishing a predictive model that used the selected biomarkers to predict the occurrence of cervical cancer in early stages. Totally 140 cervical cancer patients (stage I and II) and 140 healthy controls were recruited in the biomarker discovery phase. The miRNA expression levels and protein levels in these blood samples were measured and analyzed. The clinical characteristics of participants (e.g., age and sex) were also collected with consent forms (Table 1). Using these data, we formed a training dataset, developed a machine-learning model, and applied 10-fold cross-validation to determine its sensitivity and specificity. Next, we aimed to evaluate the applicability of the biomarker panel and thus designed an independent validation phase. In the validation phase, we recruited a new patient cohort consisted of 60 cervical cancer patients (stage I and II) and 60 healthy controls. We then analyzed the biomarker level in these participants and conducted a test to determine whether or not we could use the biomarker levels (i.e., miRNA expression levels and protein levels) in blood to discriminate early-stage cervical cancer patients from healthy controls. Our predictions were compared with pathological classification in order to evaluate the clinical performance of our biomarker panel.

**Table 1:** Characteristics of the Study Population.

Variables	Discovery phase, n = 280			Validation phase, n = 120		
	Cancer group, n = 140	Control group, n = 140	*p* value	Cancer group, n = 60	Control group, n = 60	*p* value
**Age, years**			0.324			0.410
≥55	22	27		9	12	
45-54	70	78		32	35	
<45	48	35		19	13	
**Marital status**			0.901			0.873
Married	140	138		59	58	
Unmarried	0	2		1	2	
**Menopausal status**			0.211			0.169
Postmenopausal	30	27		12	11	
Premenopausal	88	79		39	35	
Unknown	22	34		9	14	
**FIGO stage**			–			–
0 (CIN)	0			0		
I	56			26		
II	84			34		
III	0			0		
IV	0			0		
**Significant cardiac dysfunction**			0.832			0.767
Yes	4	2		2	1	
No	136	138		58	59	
**Hypertension**			0.127			0.165
Yes	11	2		5	1	
No	129	138		55	59	
**Neurological disease or diabetes**			0.619			0.578
Yes	1	0		0	1	
No	139	140		60	60	

### Comparative analysis of miRNA levels and protein levels in the discovery phase

**Figure 1:**
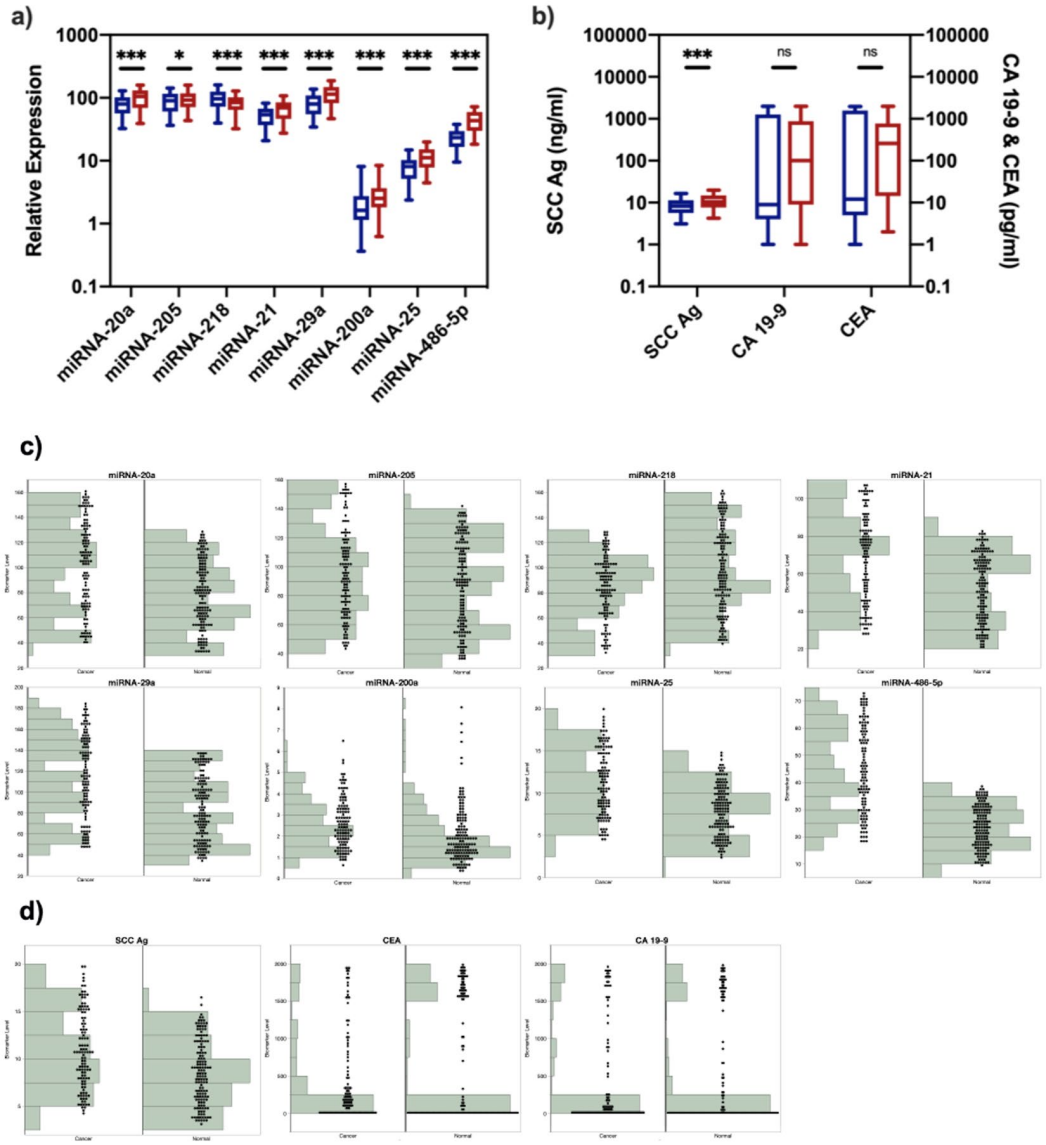
Analysis and the distribution of the expression levels of miRNA (**a,c**) and protein biomarkers (**b,d**) in training datasets. (**a**) Relative expression levels of miRNA in healthy controls (blue) and early-stage cervical cancer patients (red). **(b**) Levels of proteins in healthy controls (blue) and early-stage cervical cancer patients (red). *Indicates *p* < 0.05; ***indicates *p* < 0.001; ns indicates *p* > 0.05. (**c**) The distribution of expression levels of miRNA in healthy controls (normal) and early-stage cervical cancer patients (cancer). (**d**) The distribution of expression levels of protein biomarkers in healthy controls (normal) and early-stage cervical cancer patients (cancer).

The expression of eight miRNAs, namely miRNA-20a, miRNA-205, miRNA-218, miRNA-21, miRNA-29a, miRNA-200a, miRNA-25, and miRNA-486-5p, were measured in the blood samples of 140 cervical cancer patients (stage I and II) and 140 healthy controls in the discovery phase (Fig. 1a,c). Seven biomarkers, namely miRNA-20a, miRNA-205, miRNA-21, miRNA-29a, miRNA-200a, miRNA-25 and miRNA-486-5p, were significantly up-regulated (*p* < 0.05), while miRNA-218 was significantly down-regulated in cervical cancer patients in early stages (*p* < 0.001). Specifically, miRNA-200a, miRNA-25 and miRNA-486-5p demonstrated 1.38-fold, 1.45-fold and 1.93-fold elevated expression in cervical cancer patients in early stages (*p* < 0.001). We found that most of the miRNA showed less than 2-fold level of expression differences and we hypothesized that it was due to the early stage of cervical cancer, as several studies^25–27^ found that the cancer development at early stage did not affect miRNA expression as much as that at late stage.

Next, we measured three protein biomarkers, i.e., SCC Ag, CA19-9 and CEA, in blood samples (Fig. 1b,d). SCC Ag was significantly up-regulated in cervical cancer patients in early stages (*p* < 0.001), and demonstrated 1.28-fold elevated expression. The difference of CA19-9 and CEA levels between healthy controls and early-stage cervical cancer patients was found not significant (*p* = 0.71 and 0.17, respectively), indicating that CA19-9 or CEA alone cannot discriminate early-stage cervical cancer patients from healthy controls.

### Biomarker selection and predictive model development for discriminating cervical cancer patients from healthy controls

**Figure 2:**
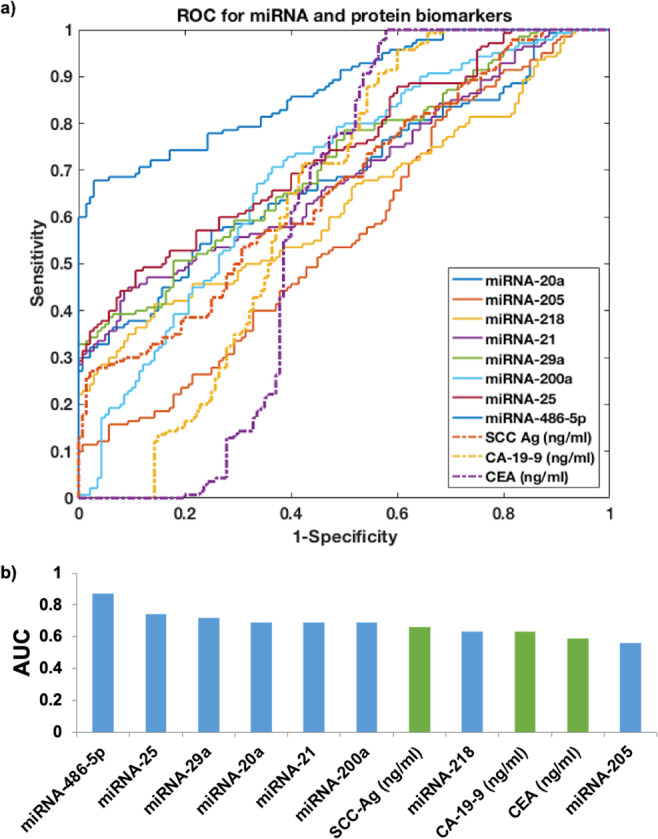
ROC curve of using single miRNA biomarkers and protein biomarkers in training datasets to predict early-stage cervical cancer. The bar plot indicates AUC value for each miRNA biomarker (blue) and protein biomarker (green).

To evaluate the discriminative power of each biomarker, we applied receiver operating characteristic (ROC) curve, followed by calculating the area under curve (AUC) value for each candidate biomarker (Fig. 2). In this study, we set an AUC value that is larger than 0.70 as our cut-off value as it suggested a decent performance for separating clinical positives with negatives. We found that the top three miRNA biomarkers were miRNA-486-5p (AUC = 0.87), miRNA-25 (AUC = 0.74) and miRNA-29a (AUC = 0.72), while the rest miRNA biomarkers did not have AUC value higher than 0.70. For the protein biomarkers, none of the biomarkers had AUC value higher than 0.70, which suggested the ineffectiveness of using single protein biomarker to discriminate cervical cancer patients in early stages and healthy controls.

We next developed a predictive model that first used three biomarkers, i.e., miRNA-29a, miRNA-25 and miRNA-486-5p, to decide whether or not a sample was from cervical cancer patients in early stages or healthy controls. We applied regression analysis by using decision tree algorithm. Decision tree is a mature machine-learning algorithm and has been widely applied in many biomedical studies^28–30^. By applying decision tree regression in our paired datasets collected in the discovery phase (i.e., biomarker level paired with the occurrence of cervical cancer of participants), we found that the sensitivity could reach 87.1% and the specificity could reach 89.3% in our model (Table 2). Next, we built another predictive model that used four biomarkers, i.e., miRNA-29a, miRNA-25, miRNA-486-5p and SCC Ag. The reason that we included SCC Ag in this model was two-fold. First, SCC Ag had a board line AUC value (AUC = 0.66), which indicated potential clinical value of discriminating cervical cancer patients in early stages from healthy controls. Second, SCC Ag was widely used in regular blood tests as well as other clinical applications. Including a protein biomarker could cover blind spots of miRNA biomarkers in cervical cancer detection. We built the new predictive model in a similar approach as that of the original model. We found that the new model with SCC Ag included as biomarker improved both sensitivity (88.6%) and specificity (92.9%). Further including other protein biomarkers failed to improve either sensitivity or specificity. Therefore, we decided to keep the model that used four biomarkers (i.e., miRNA-29a, miRNA-25, miRNA-486-5p, and SCC Ag levels in blood) as our final panel to proceed for clinical validation.

**Table 2:** The sensitivity, specificity and accuracy of different panels of miRNA and protein biomarkers in the discovery phase and validation phase.

	Panel	Sensitivity	Specificity	Accuracy
Training phase	Protein only	62.1%	62.9%	62.5%
	miRNA only	87.1%	89.3%	88.2%
	Protein+miRNA	88.6%	92.9%	90.7%
Validation phase		80.0%	96.7%	88.3%

### Biomarker validation with independent patient cohort

We designed a separate clinical validation phase to evaluate the applicability of our biomarker panel in discriminating cervical cancer patients in early stages from healthy controls. In this validation phase, we recruited totally 60 patients with cervical cancer at Stage I or II as well as 60 healthy controls. We tested the four biomarkers, namely miRNA-29a, miRNA-25, miRNA-486-5p, and SCC Ag levels, in participants’ blood and used the predictive model abovementioned to predict the occurrence of cervical cancer. We then compared our predictions with pathological classification to evaluate performance of the biomarker panel. In sum, we accurately predicted 58 of 60 healthy controls, and 48 of 60 cervical cancer patients. The sensitivity was 80.0% and the specificity was 96.7% (Table 2). The high sensitivity and high specificity, as confirmed in both the discovery phase and the validation phase, supported the conclusion that combining serum miRNA biomarkers and protein biomarkers could serve as potential, non-invasive, liquid biopsy approaches for detection of cervical cancer in early stages.

## Discussion

In this study, we found that by combining the measurement of miRNA levels and SCC Ag level in blood samples, a novel panel of biomarkers could be applied to detect cervical cancer in early stages. It is worth noticing that using single miRNA biomarker or protein biomarker did not produce a test with satisfactory sensitivity and specificity, which was the reason that we pursued for combined use of multiple biomarkers. Our multi-biomarker panel dramatically improved sensitivity and specificity of cervical cancer detection. It could provide a potentially non-invasive approach for cervical cancer detection and complement past and current liquid biopsy studies in this area. The value of this study also laid on the fact that we confirmed the clinical value of use multiple analytes in cervical cancer detection, which is attracting increasingly more interests of researchers worldwide. For instance, in a recent study by Cohen *et al*., CancerSEEK^24^ analysis measured the level of various protein biomarkers as well as multiple ctDNA mutations in sera for detection of multiple cancers such as ovarian, liver and lung cancers. CancerSEEK achieved high sensitivity in detection of cancers such as ovary and liver cancers, but was not tested for cervical cancer. Our study could bridge this gap and encourage researchers worldwide to develop and apply various types of combined biomarkers for liquid biopsy analysis.

We also would like to pinpoint that to use combined biomarkers for cervical cancer detection, it is crucial to develop machine-learning-based predictive model. Machine learning has been widely used in many areas of liquid biopsy studies to help researchers improve the performance of various biomarkers including ctDNA^31^, ctRNA^32^, proteomics^33,34^, and metabolomics^35^. When using multiple types of biomarkers in cancer detection, it often requires de-convoluting a complex system that is highly nonlinear with multi-dimensionality. Machine learning is particularly suitable for such task. However, one important note we want to make is that overfitting issue, which often leads to falsely high sensitivity and high specificity, should be taken into consideration when developing machine-learning models. For example, we specifically designed the biomarker validation phase, in which we tested our method in a new cohort of participants. We found that the sensitivity and specificity remained at a level that was similar to that of the discovery phase. This indicated that our method successfully avoided the overfitting issue.

We also would like to point out a few limitations of this study. First, the samples in this study were collected from participants from China only. Race-associated biomarkers could exist and bias the conclusion. To this end, we are currently collaborating with several researchers in U.S. and India to further evaluate the applicability of our biomarker panel in different races. Second, the purpose of this study is mainly focusing on discovering and validating a panel of biomarkers for cervical cancer detection in early stages. Investigating the biomolecular mechanisms of these biomarkers in cervical cancer development is beyond the scope of our study here. With this purpose in mind, we mostly replied on previous studies on cervical cancer biomarkers and solicited the biomarkers as suggested, i.e., protein biomarkers (CEA, SCC Ag and CA19-9)^10–14^ and miRNA biomarkers (miRNA-20a, miRNA-205, miRNA-218, miRNA-21, miRNA-29a, miRNA-200a, miRNA-25, miRNA-486-5p)^9,20–22^, to detect early-stage cervical cancer. There certainly exists the possibility of missing some biomarkers that are not included in this study but effective in detection of cervical cancer in early stages. We will continue to explore more biomarkers in cervical cancer detection.

To conclude, we discovered a biomarker panel that combined the measurement of serum protein level and miRNA levels for accurate detection of cervical cancer patients in early stages. Our study proves the concept of using multiple analytes of blood for improved sensitivity and specificity in liquid biopsy studies.

## Methods

### Samples and clinical characteristics

This study was conducted with approval by Institutional Review Board of Jinan People’s Hospital and in adherence to the Declaration of Helsinki Principles. Informed consent was obtained from all participants (all above 18 years of age) prior to use any clinical material for research purposes. Table 1 listed the clinical information of the samples. In brief, this study was divided into two phases: a biomarker discovery phase and a validation phase. A total of 140 cervical cancer patients, together with 140 non-cervical cancer people (i.e., healthy controls), were recruited in the discovery phase from April 2nd, 2015 to January 8th, 2018 in Jinan People’s Hospital. Following the discovery phase, we used similar approaches to recruit totally 60 cervical cancer patients and 60 healthy controls in the validation phase from February 6th, 2018 and January 10th, 2019. We only included cervical cancer patients that were confirmed with diagnosis of cervical cancer via histology or cytology. We excluded those patients that were treated with chemotherapy or radiotherapy in the past 30 days or diagnosed with other malignancies prior to blood collection. A total of 2 mL peripheral blood samples were collected using tubes without anti-coagulant, followed by separating sera within half an hour after sample collection and storage at −20 °C for analytics.

### miRNA extraction, cDNA synthesis and quantification

The following procedures were applied to extract miRNA, synthesize cDNA and quantify miRNA level, in both the biomarker discovery phase and the validation phase. In sum, 250 μl of whole blood was collected. We centrifuged the blood samples at 1,200 × g at 4 °C for 20 min, followed by passing through a 13-mm serum filter (Thermo Fisher Scientific Inc.). The supernatant was used for total RNA isolation. The similar approach has been used in previous studies for extracting circulating microRNAs^21^. Trizol LS reagent (Invitrogen, Carlsbad, CA, USA) was used as the reagent for total RNA extraction by following the manufacturer’s protocol. To determine the quality and quantity of RNA, we used Nanodrop spectrophotometer (ND-1000, Nanodrop Technologies) for measurement. We also determined RNA integrity by gel electrophoresis. Next, we synthesized cDNA by using miRNA as templates. This step was conducted by using TaqMan microRNA reverse transcription kit (Takara Biotechnology, Co., Ltd.). We then performed reverse transcription with the Invitrogen SuperScript III Reverse Transcriptase kit by using miRNA-specific primers (Table 3). PrimeScript RT Master Mix (Takara Biotechnology, Co., Ltd.) was performed in triplicate in qPCT on a Bio-Rad CFX96 real-time PCR machine (Bio-Rad Laboratories, Inc., Hercules, CA, USA). We chose U6, miRNA-16 and miRNA-25 as an internal control for normalization of miRNA levels. U6, miRNA-16 and miRNA-25 have been previous reported to be stably expressed and used as internal controls in miRNA expression studies^20,22,36^. The cycling parameters for miRNA were as follows: 95 °C for 30 sec, followed by 40 cycles of 95 °C for 5 sec and 60 °C for 30 sec. Finally, we quantified the miRNA level by using the ΔCt method that was previously described^37–39^. In short, the miRNA level = 2^ ΔCt multiplying 1000, where ΔCt was the difference between Ct value of the miRNA of interest to Ct value of internal controls.

**Table 3:** Primers used in this study.

Primer name	Sequence
MiR-20a_F	TACGATAAAGTGCTTATAGTGCAGGTAG
MiR-20a_R	GTCCTTGGTGCCCGAGTG
MiR-205_F	TCCTTCATTCCACCGGAGTCTG
MiR-205_R	GTCCTTGGTGCCCGAGTG
MiR-218_F	CGGAATTCATGGGCAAAGGA
MiR-218_R	GTCCTTGGTGCCCGAGTG
MiR-21_F	TAGCTTATCAGACTGATGTTGA
MiR-21_R	GTCCTTGGTGCCCGAGTG
MiR-29a_F	TAGCACCATCTGAAATCGG
MiR-29a_R	GTCCTTGGTGCCCGAGTG
MiR-200a_F	AGTGGGGCTCACTCTCCAC
MiR-200a_R	GTCCTTGGTGCCCGAGTG
MiR-25_F	ATTGCACTTGTCTCGGTCTG
MiR-25_R	GTCCTTGGTGCCCGAGTG
MiR-486-5p_F	ACACTCCAGCTGGGTCCTGTACT
MiR-486-5p_R	GTCCTTGGTGCCCGAGTG
MiR-16-5p_F	CTGCAGGGATCTAGGATTACAAGT
MiR-16-5p_R	GCCGGCCATTATGCACATACCAGT
MiR-25-5p_F	GCAGCATTGCACTTGTCTCG
MiR-25-5p_R	AGTGCAGGGTCCGAGGTATTC
U6_F	ATTGGAACGATACAGAGAAGATT
U6_R	GGAACGCTTCACGAATTTG

### Analysis of protein levels in blood samples

We applied Bioplex 200 platform (Biorad, Hercules CA) together with luminex bead based immunoassays (Millipore, Bilerica NY) to determine the level of CA19-9 and CEA in serum according to manufacturer’s instructions. In brief, we diluted serum samples for 10-100 times and loaded standards (provided by vendor) and serum samples in duplicates. We used HCCBP1MAG-58K panel to analyze CA19-9 and CEA, and calculated the concentrations of CA19-9 and CEA by referring to the corresponding standard curves using the software of Bioplex Manager. To analyze SCC Ag, we strictly followed the protocol of Architect i2000 (Abbott Laboratories, Abbott Park, IL, USA) for measurement. The standard curve and cut-off values were prepared according to manufacturer to calculate the final concentration of SCC Ag.

### Statistical and machine learning analysis

We used GraphPad Prism 5.0 software (San Diego, CA) for all the following statistical analysis. The different level of mRNA and proteins in blood between cervical cancer patients and healthy controls was evaluated using Student’s t test. As suggested by multiple studies, we considered *p* < 0.05 as statistically significant. Next, we calculated the area under the curve (AUC) by conducting receiver operating characteristic (ROC) analysis. In general, biomarker with AUC > 0.70 was considered to be discriminative and further selected for machine learning analysis. We then selected decision tree algorithm as our machine learning classifier, and used the ‘fitctree’ function in Statistics and Machine Learning Toolbox of in MATLAB^TM^ software for regression modeling. Decision tree is a mature machine-learning algorithm and has been widely applied in many biomedical studies^28–30^. In case overfitting issue existed, we applied k-fold cross-validation where k was chosen as 10 in this study. After training the classifier with data from biomarker discovery phase, we then applied it to the datasets in the independent clinical validation phase. We used RNA and protein levels as the features for the classifier, and used ‘predict’ function in Statistics and Machine Learning Toolbox in MATLAB^TM^ software to compute the occurrence of cervical cancer. We compared our predictions with diagnosis and reported both sensitivity and specificity as criteria of performance.

## Data Availability

All data are available within the Article.
